# Maladie de kaposi classique botriomycome-like: un piège diagnostic

**DOI:** 10.11604/pamj.2014.17.283.3877

**Published:** 2014-04-15

**Authors:** Hanae Bouzidi, Salim Gallouj, Nissrine Amraoui, Fatima Zahra Mernissi, Taoufiq Harmouch

**Affiliations:** 1 Service de Dermatologie-Vénérologie, CHU Hassan II, Fès, Maroc; 2 Service d'Anatomopathologie, CHU Hassan II, Fès, Maroc

**Keywords:** Maladie de kaposi, botriomycome, diagnostic, Kaposi disease, botriomycoma, diagnosis

## Abstract

La maladie de kaposi (MK) botriomycome-like et une variante clinique et anatomopathologique rare de la MK, rapportée aussi bien dans la forme classique et épidémique de la MK. C'est une entité difficile à diagnostiquer car ses caractéristiques cliniques et histologiques englobent à la fois celles du botriomycome et de la MK. En plus du contexte clinique l’étude histologique et immunohistochimique restent primordiales pour établir son diagnostic. Nous rapportons un cas de MK botriomycome-like assez particulier pas sa localisation et son siège unique.

## Introduction

La maladie de kaposi (MK) botriomycome-like et une variante clinique et anatomopathologique rare de la maladie de kaposi, une maladie proliférative à double composante vasculaire et fibroblastique induite par le huitième virus de l'herpès humain HHV-8. Elle se manifeste généralement dans sa forme classique ou dite également méditerranéenne par des papulo-nodules angiomateux siégeant au niveau des membres inferieurs des sujets âgés de plus de 60 ans sur un terrain de lymphoedème. La localisation céphalique inhabituelle dans cette forme d'autant pus que l'aspect botriomycome-like.

## Patient et observation

**Figure 1:**
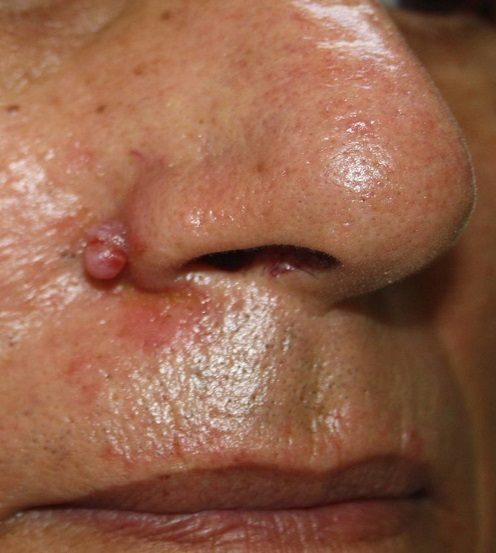
Nodule érythémateux de 6mm au niveau du sillon naso-génien

**Figure 2:**
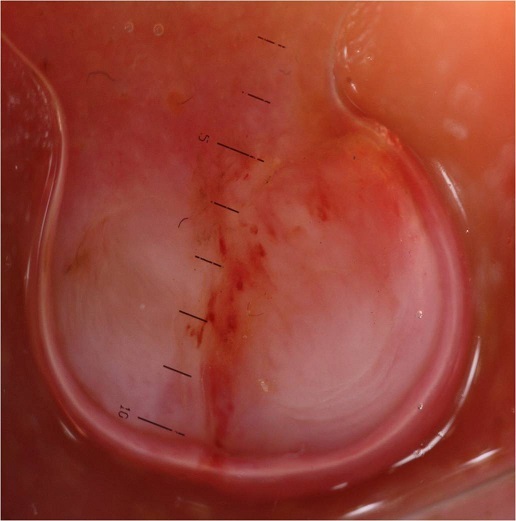
Examen au dermoscope à immersion: aires laiteuses traversées par des vaisseaux et spots hémorragiques et entourées d'une collerette périphérique

**Figure 3:**
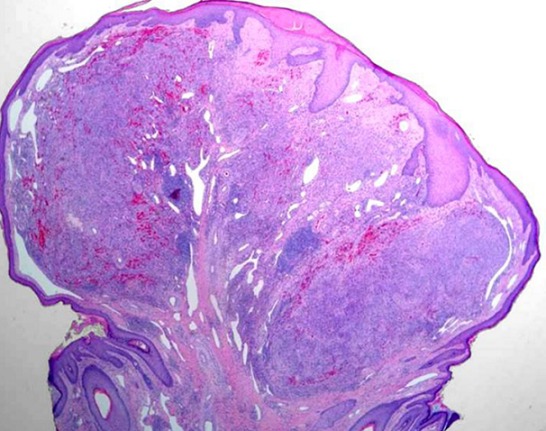
Coupe histologique (HE): masse exophytique enveloppée par une collerette épidermique avec présence de cellules fusiformes et extravasation de cellules sanguine

Mr O.A âgé de 70 ans, originaire du Maroc, consultait pour un nodule érythémateux unique de 6mm de grand axe au niveau du sillon naso-génien droit ayant apparu depuis 2 mois. Le patient rapportait l'augmentation du volume de la lésion après un traumatisme local (Figure 1). L'examen au dermoscope à immersion a objectivé des aires laiteuses traversées par des vaisseaux linéaires et des spots hémorragiques et entourées d'une collerette blanchâtre périphérique (Figure 2). L'aspect clinique et dermoscopique évoquaient en premier un botriomycome. La biopsie exérèse avec étude immunohistochimique était en faveur d'une maladie de kaposi (Figure 3) avec une masse exophytique enveloppée par une collerette épidermique et présence de cellules fusiformes et extravasation de cellules sanguines, l’étude immunohistochimique a objectivé un marquage CD 31 et CD 34 positifs, le marquage LAN-1 (antigène nucléaire de latence-1): un anticorps monoclonal contre le HHV-8 n'a pas été réalisé étant non disponible dans notre centre hospitalier. Par ailleurs, le patient a bénéficié d'un bilan paraclinique radiologique n'ayant pas objectivé d'autres localisations avec une sérologie HIV négative. Il a été classé comme forme méditerranéenne. Le patient n'a pas présenté de nouvelles lésions avec un recule de 1 an.

## Discussion

La MK est une maladie proliférative multifocale, d'expression cutanée et viscérale impliquant diverses cellules mésenchymateuses [1, 2]. Elle est classée en 4 formes épidémiologiques: 1) MK classique, touchant le plus souvent les hommes âgés originaires de l'Europe centrale, l'Europe de l'Est et les méditerranéens avec une prédominance chez la population juive; 2) MK endémique (Afrique subsaharienne); 3) MK iatrogénique associée à une immunosuppression; 4) MK épidémique (associée au SIDA/ HIV) [3]. La MK classique se présente habituellement sous forme de macules violines et érythémateuses qui évoluent lentement vers des plaques et des nodules angiomateux. Ces lésions sont habituellement localisées au niveau des extrémités distales [1]. La MK endémique atteint une population plus jeune avec une évolution rapide, localement agressive et s'accompagne souvent d'une extension aux ganglions lymphatiques et d'une atteinte viscérale. La maladie est alors assez souvent mortelle [4]. La MK Iatrogénique survient chez des sujets soumis à des traitements immunosuppresseurs au long cours, dans le cadre ou non de transplantation d′organes, se manifeste par plusieurs lésions cutanées sur les extrémités distales et suit une évolution bénigne [5, 6]. La MK associée au SIDA/HIV se présente sous des formes cutanées et muqueuses extensives avec atteinte ganglionnaire et viscérale et peut conduire rapidement au décès [7].

La localisation céphalique étant très rare dans la MK classique et d'emblée associée à une atteinte des extrémités. Notre cas est non seulement caractérisé par sa localisation particulière et unique mais aussi par l'aspect clinique et dermoscopique prêtant confusion avec le botriomycome, une tumeur bénigne ne nécessitant pas un bilan d'extension ou un suivi particulier. Cet aspect a été déclenché par un traumatisme ce qui témoigne de l'incrimination du phénomène de koebner même dans cette pathologie.

La MK botriomycome-like a été rapportée chez des patients à la fois HIV séropositifs et séronégatifs dans le cadre de la MK classique [8, 9, 10]. Dans un rapport, la MK botriomycome-like a été d′abord prise pour un botriomycome [8]. Dans un autre rapport, la MK botriomycome-like se situait sur les mains de trois patients, une localisation où les botriomycomes sont généralement trouvés [10].

Sur le plan histologique, le botriomycome présente généralement sous forme d'une prolifération capillaire disposée en lobules formant une touffe issue d'un pédicule vasculaire émergeant des plans cutanés profonds. Un recouvrement épidermique aminci contrastant avec la présence d'une collerette épidermique invaginée à la périphérie de la lésion. Un infiltrat inflammatoire d'accompagnement, au départ absent, apparaît de manière variable et inconstante dans les suites de l’érosion [11].

Les quatre formes épidémiologiques de la MK présentent des particularités cliniques et évolutives différentes mais leurs caractéristiques histologiques sont communes. Avec une évolution histologique progressive à partir du stade maculeux arrivant à la plaque et finalement au stade nodulaire. Le diagnostic histopathologique de la MK est basé sur la présence simultanée de structures vasculaires, de cellules fusiformes, d’éléments lympho-plasmocytaires et de dépôts ferriques [12]. Il ne se présente pas de différences majeures selon la forme clinique ou la localisation (cutanée ou viscérale) des lésions; Cependant, dans les dernières décennies, il ya eu une sensibilisation croissante vers un large spectre histologique [12, 13].

Des observations récentes ont conduit à un nouveau classement des variantes histologiques de la MK: 1) variantes liées à la progression de la maladie; 2) variantes évoquées dans la littérature ancienne KS; 3) variantes récentes; 4) variantes liées aux résultats du traitement [12]. La MK botriomycome-like est classée dans la " variante récente de la MK " et peut être très difficile à distinguer du botriomycome vu les caractéristiques histologiques qui se chevauchent, comme la collerette épidermique résultant de la proéminence nodulaire, l'ulcération et l′inflammation et la prolifération lobulaire de capillaires [13]. En effet les petites lésions nodulaires ou micronodulaires superficielles de la MK peuvent être protubérantes et susciter ainsi le développement d′une collerette épidermique périphérique. Les lésions traumatisées peuvent subir une ulcération et une inflammation, et donc préter confusion avec le botriomycome. Pour compliquer encore les choses, le botriomycome peut se développer sur zones kaposiformes [12, 14, 15].

L′analyse immunohistochimique avec des anticorps des cellules musculaires lisses (SMA) et le Facteur VIII (FVIII), souvent retrouvées dans les péricytes du botriomycome et les cellules endothéliales matures, respectivement, peut aider à faire la distinction entre les botriomycomes et la MK. Dans la MK, les péricytes et les cellules endothéliales matures ne sont pas observées [11]. Les Marqueurs endothéliaux, comme le CD31, le CD34 et les marqueurs lymphatiques D2-40 marquent les cellules fusiformes des lésions nodulaires du Kaposi [11, 13]. Cependant, l′expression simultanée des deux marqueurs du botriomycome et du kaposi peut se produire. L'exemple d'un cas rapporté de MK botriomycome-like ayant démontré l'expression de SMA, FVIII, CD31, CD34, dans ce cas c'est immunomarquage de l'HHV- 8 LNA qui a confirmé le diagnostic [10]. Sa sensibilité et sa spécificité pour la MK a été démontrée dans plusieurs études [14, 16–18].

## Conclusion

La MK peut mimer cliniquement et histologiquement un botriomycome, représentant ainsi un piège diagnostique. La MK botriomycome-like est très rare, ce qui accroît la difficulté diagnostique de cette entité et l'emplacement sur le visage augmente le défi diagnostique. L’étude immunohistochimique reste le gold standard pour établir le diagnostic.
